# The American Society of Microscopists

**Published:** 1889-09

**Authors:** 


THE AMERICAN SOCIETY OF MICROSCOPISTS held a very successful meeting in this city, August 20–23, 1889. Dr. George E. Fell, of Buffalo, was elected president for the ensuing year.

